# Metagenomic Analysis of Rhizospheric Bacterial Community of Citrus Trees Expressing Phloem-Directed Antimicrobials

**DOI:** 10.1007/s00248-024-02408-w

**Published:** 2024-07-15

**Authors:** Leandro Alberto Núñez-Muñoz, Martín Eduardo Sánchez-García, Berenice Calderón-Pérez, Rodolfo De la Torre-Almaraz, Roberto Ruiz-Medrano, Beatriz Xoconostle-Cázares

**Affiliations:** 1 Departamento de Biotecnología y Bioingeniería, Centro de Investigación y de Estudios Avanzados, Av. Instituto Politécnico Nacional 2508, Col. San Pedro Zacatenco, 07360 Mexico City, Mexico; 2 Facultad de Estudios Superiores Iztacala, Universidad Nacional Autónoma de México, 54090 Mexico City, Estado de México Mexico; 3 Centro de Investigación y de Estudios Avanzados, Programa de Doctorado Transdisciplinario en Desarrollo Científico y Tecnológico Para La Sociedad, Av. Instituto Politécnico Nacional 2508, Col. San Pedro Zacatenco, 07360 Mexico City, Mexico

**Keywords:** Phloem-directed antimicrobials, Microbial diversity, Transgenic citrus, HLB mitigation

## Abstract

Huanglongbing, also known as citrus greening, is currently the most devastating citrus disease with limited success in prevention and mitigation. A promising strategy for Huanglongbing control is the use of antimicrobials fused to a carrier protein (phloem protein of 16 kDa or PP16) that targets vascular tissues. This study investigated the effects of genetically modified citrus trees expressing *Citrus sinensis* PP16 (CsPP16) fused to human lysozyme and β-defensin-2 on the soil microbiome diversity using 16S amplicon analysis. The results indicated that there were no significant alterations in alpha diversity, beta diversity, phylogenetic diversity, differential abundance, or functional prediction between the antimicrobial phloem-overexpressing plants and the control group, suggesting minimal impact on microbial community structure. However, microbiota diversity analysis revealed distinct bacterial assemblages between the rhizosphere soil and root environments. This study helps to understand the ecological implications of crops expressing phloem-targeted antimicrobials for vascular disease management, with minimal impact on soil microbiota.

**Supplementary Information:**

The online version contains supplementary material available at 10.1007/s00248-024-02408-w.

## Introduction

Citrus Huanglongbing (HLB), also known as citrus greening, is a major threat to the global citrus industry, causing severe economic losses annually. HLB is caused by *Candidatus* Liberibacter asiaticus (*C*Las), *Ca*. L. americanus, and *Ca*. L. africanus, with *C*Las being the most prevalent. *C*Las is a bacterium restricted to the sieve tubes which blocks the transport of photoassimilates and other molecules, thus causing vascular obstruction [1]. HLB symptoms include shoot yellowing, blotchy mottled leaves, corky veins, malformed and discolored fruits, premature fruit drop, root loss, and eventual tree death [2]. In the advanced stages of HLB infection, there is a decrease in plant carbohydrate metabolism, resulting in severe impairment of root growth owing to reductions in starch transport and accumulation [3]. Currently, there are no proven strategies to prevent or eradicate HLB. HLB control strategies are focused on the pathogen, host, or vector. Pathogen-targeting methods include inhibition of essential *C*Las proteins, use of antibiotics, antimicrobials, immunity-inducing compounds, and thermotherapy. Host-targeting approaches enhance immunity via advanced nutrition programs, transgenic varieties, resistant citrus-related germplasms, soil conditioners, or inducing systemic acquired resistance. Vector-targeting strategies encompass chemical and biological control and eradicating HLB-infected trees [4–8]. Additional novel strategies include antimicrobial compounds such as 2S albumin, nano-zinc oxide, Type 1 lipid transfer proteins, TcyA inhibitors, and PRpnp proteins [9–12]. However, the efficacy of these strategies varies under field conditions and remains debatable.

Transcriptome and proteome analyses of the plant vasculature, specifically the phloem sap, have revealed a large diversity of RNAs and proteins that participate in phloem maintenance, cell signaling, stress response, innate immunity, response to pathogen attack, flowering, and tuberization [13]. Among these vascular proteins, 16 kDa phloem protein (PP16) can enter the phloem and facilitate the non-specific translocation of proteins and RNA into the phloem translocation stream [14]. Therefore, PP16 from *Cucurbita maxima* (CmPP16) and *Citrus sinensis* (CsPP16) have been employed as vascular systemic transporters of antimicrobials, including human lysozyme and beta-defensins, to mitigate HLB infection [15, 16]. However, the ecological implications of this approach, particularly for soil microbiome diversity, remain unclear. We hypothesized that this strategy could effectively control *C*Las while minimizing the impact of antimicrobials on non-target microorganisms such as beneficial soil and root microbiota.

The soil microbiome is pivotal for plant development, ecosystem functioning, and overall plant health. Beneficial soil microorganisms play key roles in nutrient cycling (e.g., nitrogen fixation or phosphate solubilization), disease suppression, and enhancement of plant growth, as well as in conferring tolerance to stress [17]. Nonetheless, HLB infection modifies root-associated bacterial and fungal communities, destabilizing microbial equilibrium and inducing dysbiosis. For example, *C*Las-infected roots exhibited a relative abundance increase of *Amycolatopsis*, *Sphingopyxis*, *Chryseobacterium*, *Flavobacterium*, *Ralstonia*, *Stenotrophomonas*, *Duganella*, and *Streptacidiphilus*, contrasting with a relative abundance decline of *Rhizobium* [18]. Other studies have revealed significant microbial changes in the endosphere and rhizosphere of HLB-affected citrus plants [19]. In addition, at early disease stages, a relative abundance increase of keystone and beneficial species occurred. Trees with slow disease progression exhibited higher beneficial microbe abundances, whereas non-survivors or highly symptomatic trees were enriched with pathogenic or antibiosis-associated microbes, leading to saprophytes proliferation in advanced infection stages [20].

Genetically modified (GM) plants offer economic benefits but raise concerns about biosafety and ecological compatibility, including effects on microbial communities. Evidence shows variable impacts: *Arabidopsis thaliana* overexpressing thionin showed no significant changes [21], while GM banana expressing hrap and pflp proteins maintained stable bacterial communities [22]. Conversely, GmMYB10 in *Glycine max* altered microbial communities, increasing *Bacillus*, *Aspergillus*, and *Talaromyces* [23]. Bt crops showed mixed effects on soil microbial biomass and bacterial communities, with other studies reporting minimal impact in bacterial communities [24–26]. Gene overexpression in other GM plants altered rhizospheric bacterial composition, and EPSPS-transgenic soybeans exhibited transient effects on nitrogen-fixing bacteria [27, 28]. These studies suggest the importance of maintaining the structure and diversity of microbial communities as they have beneficial effects on crops.

Consequently, it is essential to investigate the potential effects of GM citrus trees expressing phloem-directed antimicrobials on the soil microbiome diversity. Hence, this study aimed to characterize the microbial communities of citrus plants expressing CsPP16-antimicrobial fusion and evaluate its potential ecological implications. The findings could guide future research and practical applications in HLB management, enhancing sustainable citrus production.

## Methods

### Site Description and Sampling

The study was conducted in an open-field citrus plantation in Tecomán, Colima, Mexico (18° 56′ 5.02″ N, 103° 55′ 34.88″ W), located about 10 km from the Pacific coast at 33 m above sea level with a warm subtropical climate. The experimental field consisted of GM *Citrus aurantifolia* trees expressing human lysozyme C (NP_000230.1), human β-defensin-2 (NP_001192195), a combined antimicrobial treatment, and a control group. The antimicrobials were expressed as fusion proteins with CsPP16 (XP_006486477), and the *C. aurantifolia* transgenic scions were grafted onto certified (HLB-free) *Citrus volkameriana* rootstocks and arranged randomly in the experimental field [16]. Citrus trees expressing antimicrobials and control plants were challenged to populations of the insect vector *Diaphorina citri* infected with *C*Las. HLB infection was detected by *C*Las quantification in citrus plants [16]. Soil and root sampling was conducted 3 months after planting. Root surfaces were disinfected as previously described [29]. Composite samples obtained from four different trees per replicate were collected using the quartering method and stored at − 80 °C until DNA extraction. Each treatment comprised 3 composite rhizospheric soil samples (12 in total), along with 4 control plant root composite samples.

### Genomic DNA Extraction

DNA extraction was performed from 100 mg of rhizospheric soil or roots using the PowerSoil DNA Isolation Kit (MO BIO Laboratories Inc., Carlsbad, CA, USA) according to the manufacturer instructions with the following modification: an agitation step of 15 min at 50 Hz using a TissueLyser (QIAGEN, Hilden, Germany) after adding C1 solution. Subsequently, DNA integrity was assessed using 0.8% agarose gel electrophoresis and DNA concentration was determined using a NanoDrop One spectrophotometer (Thermo Fisher Scientific, Waltham, MA, USA).

### Library Construction

Barcoded amplicons containing the V3-V4 variable region of 16S rRNA gene were amplified by PCR using the 357F (CTCCTACGGGAGGCAGCAG) and 783R (CTACCAGGGTATCTAATCCTG) primers [30, 31]. PCR reactions were performed in 50 µL containing 1X PCR buffer, 2 mM MgCl_2_, 0.2 μM of each barcoded primer, 0.2 mM of each dNTP, 1.25 U of recombinant Taq DNA polymerase (Thermo Scientific, Waltham, MA, USA), and 10 ng of genomic DNA. The PCR program included denaturation at 95 °C for 5 min, followed by 30 cycles of denaturation at 94 °C for 30 s, annealing at 62 °C for 15 s, extension at 72 °C for 15 s, and a final extension at 72 °C for 10 min using a GeneAmp PCR System 2700 (Applied Biosystems, Waltham, MA, USA). Library size and concentration were assessed using the 2100 Bioanalyzer System (Agilent, Santa Clara, CA, USA). PCR products were purified using the Wizard SV Gel PCR Clean-Up System (PROMEGA, Madison, WI, USA). High-throughput sequencing and further processing were carried out using the Ion One Touch 200 template kit V2 DL, Ion One TouchTM 2 System, Ion OneTouch™ ES, Ion 316™ Chip v2, Ion PGM™ Sequencing 200 Kit v2, and Ion Torrent PGM System (Life Technologies, CA, USA) at the “Reference and Support Laboratory for Genome, Transcriptome, and Microbiome Characterization” (Genetics and Molecular Biology Department, CINVESTAV, Mexico City, Mexico). Raw sequences were filtered, demultiplexed, and exported using the Ion Torrent PGM software (Life Technologies, CA, USA).

### Bioinformatic and Statistical Analysis

Quality control was performed using FastQC and MultiQC programs. Microbiome bioinformatic analyses were performed using QIIME 2 2023.2. Default parameters were used for all plugins and software unless stated otherwise. Adapters were removed using cutadapt with *qiime cutadapt trim-paired* (–p-cores 4, –p-front-f CTCCTACGGGAGGCAGCAG, –p-front-r CTACCAGGGTATCTAATCCTG). DADA2 was called through *qiime dada2 denoise-paired* (–p-trunc-len-f 260, –p-trunc-len-r 230, –p-trim-left-f 17, –p-trim-left-r 17, –p-pooling-method pseudo, –p-chimera-method pooled) to denoise, dereplicate, and filter chimeras. For classifier training, the 357F and 783R amplicon regions were extracted from the QIIME-compatible Greengenes 13_8 database at 97% yield using *qiime feature-classifier extract-reads* (–p-f-primer CTCCTACGGGAGGCAGCAG, –p-r-primer CTACCAGGGTATCTAATCCTG, –p-min-length 50, –p-max-length 500). The classifier model was then fitted and trained using *qiime feature-classifier fit-classifier-native-bayes* and *qiime feature-classifier classify-sklearn* commands (–p-confidence 0.97). Reads assigned to mitochondria or chloroplasts were removed using *qiime taxa filter-table qiime taxa filter-seqs* (–p-exclude mitochondria, chloroplast). Subsequently, a phylogenetic tree was then constructed using fasttree and mafft alignments with *qiime phylogeny align-to-tree-mafft-fasttree*.

Statistical analyses were performed in R (v. 4.1.2) using qiime2R, phyloseq, tydiverse, vegan, microbiome, DESeq2, ggpicrust2, and picante packages. Relative abundance analysis was performed using phyloseq, whereas differential abundance analysis was conducted with DESeq2, with the Benjamini–Hochberg False Discovery rate (FDR) *p*-value adjustment and a Cook distance cutoff of 0.99.

Observed Amplicon Sequence Variants (ASVs), ACE, Chao1, Fisher, Shannon, and Simpson alpha diversity indices were estimated using the phyloseq *estimate_richness* function, and statistical comparisons were performed using the Kruskal–Wallis non-parametric test and pairwise Wilcoxon tests. Weighted UniFrac analysis was conducted using the *UniFrac* function from the picante package, and rarefied data normalized to the minimum reads per sample.

To evaluate beta diversity, principal coordinate analysis (PCoA) was conducted using the phyloseq *plot_ordination* function, followed by permutational multivariate analysis of variance (PERMANOVA) using the *adonis2* function from the vegan package. Additionally, for the rhizospheric soil bacterial communities of the plants, treatment similarities were assessed by analysis of similarities (ANOSIM) using the vegan package. For phylogenetic diversity estimation using Faith index, data were rarefied to the minimum sample read counts and the index was calculated using the *pd* function of the picante package. Predictive functional analysis of microbial communities in the rhizosphere of plants overexpressing phloem-targeted antimicrobials was conducted using PICRUSt2 (Phylogenetic Investigation of Communities by Reconstruction of Unobserved States), using the *picrust2_pipeline.py* function (Douglas et al., 2020). This approach enabled inference of the relative abundance of potential metabolic pathways from the 16S rRNA gene sequence profiles. Pathway annotation was performed using the *pathway_annotation* function in the MetaCyc database of the ggpicrust2 package for functional annotation. Subsequently, a differential abundance analysis of predictive functional pathways (DA) was performed using the *pathway_daa* function by applying the ALDEx2 method for high-throughput sequencing data analysis, FDR *p*-value adjustment, and ALDEx2 Welch’s test in the ggpicrust2 package. In the differential abundance graphs, only those metabolic pathways with an adjusted *p*-value < 0.05 and log2 (fold change) values exceeding 1 or less than − 1 were shown.

## Results

A total of 3,263,355 raw sequences were generated from 16 samples. Following trimming, filtering, denoising, and removal of chimeras, the dataset was refined to 2,591,308 reads. Among these, 1,527,316 sequences (58.93% of the filtered reads and 46.8% of the raw sequences) were assigned to the Domain Bacteria (Table S1). No sequences were assigned to the Domain Archaea. Furthermore, 9668 ASVs were identified and classified into 39 phyla, 111 classes, 159 orders, 199 families, and 242 genera.

### Differential Abundance Analysis

**Fig. 1:**
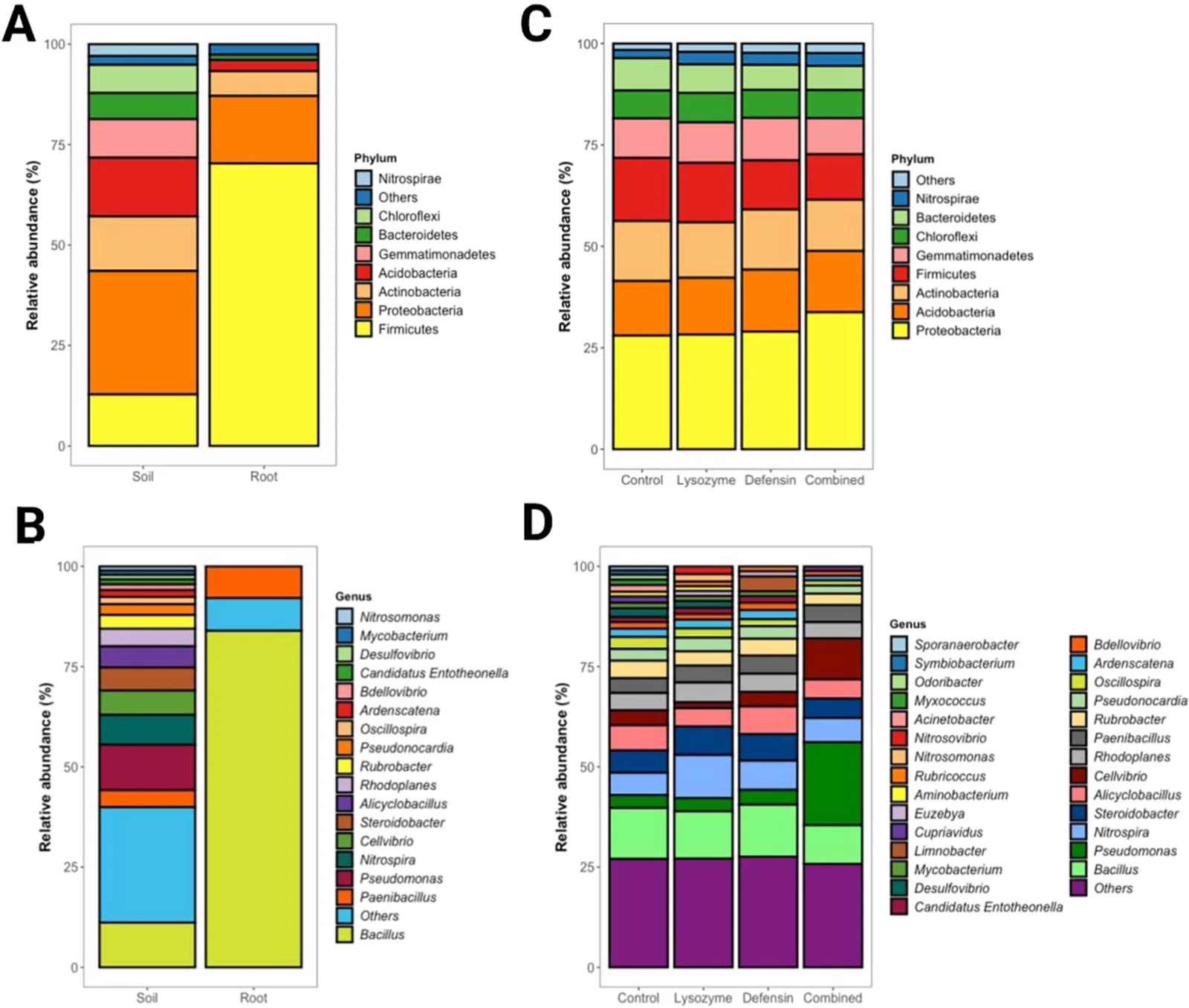
Comparative analysis of bacterial community abundance in *C. aurantifolia.* Relative abundance of bacterial communities associated with the control rhizospheric soil and roots of *C. aurantifolia*, categorized at the phylum (**A**) and genus (**B**) levels. The relative abundances in the rhizospheric soil of *C. aurantifolia* plants overexpressing phloem-directed antimicrobials, grouped at the phylum (**C**) and genus (**D**) levels

For the abundance analysis, the data were categorized into high and low taxonomic levels. In the rhizosphere soil, 33 phyla were identified at the phylum level: Proteobacteria (30.67%), Acidobacteria (14.66%), Actinobacteria (13.59%), Firmicutes (12.87%), Gemmatimonadetes (9.54%), Chloroflexi (7.02%), Bacteroidetes (6.54%), Nitrospirae (2.94%), and other 25 phyla with abundances of less than 1% (2.15%). In the roots of control plants, 22 phyla were detected with predominance of Firmicutes (70.33%), Proteobacteria (16.79%), Actinobacteria (6.15%), and Acidobacteria (2.8%). The remaining 18 phyla accounted for a relative abundance of 2.59% (Fig. 1A).

The most abundant genera in the rhizosphere soil were *Pseudomonas* (11.30%), *Bacillus* (11.16%), *Nitrospira* (7.40%), *Cellvibrio* (6.11%), *Steroidobacter* (5.75%), *Alicyclobacillus* (5.31%), *Rhodoplanes* (4.36%), *Paenibacillus* (4.25%), *Rubrobacter* (3.42%), *Pseudonocardia* (2.67%), *Oscillospira* (1.82%), *Ardenscatena* (1.74%), *Bdellovibrio* (1.43%), *Candidatus* Entotheonella (1.23%), *Desulfovibrio* (1.15%), *Mycobacterium* (1.05%), and *Nitrosomonas* (1.00%). Genera with abundances < 1% accounted for 28.85% encompassing 215 genera. In the roots, the most abundant genera were *Bacillus* (83.99%) and *Paenibacillus* (7.86%), while the remaining genera accounted for 8.15% and included *Candidatus* Liberibacter (0.56%) among other 103 genera (Fig. 1B).

**Fig. 2:**
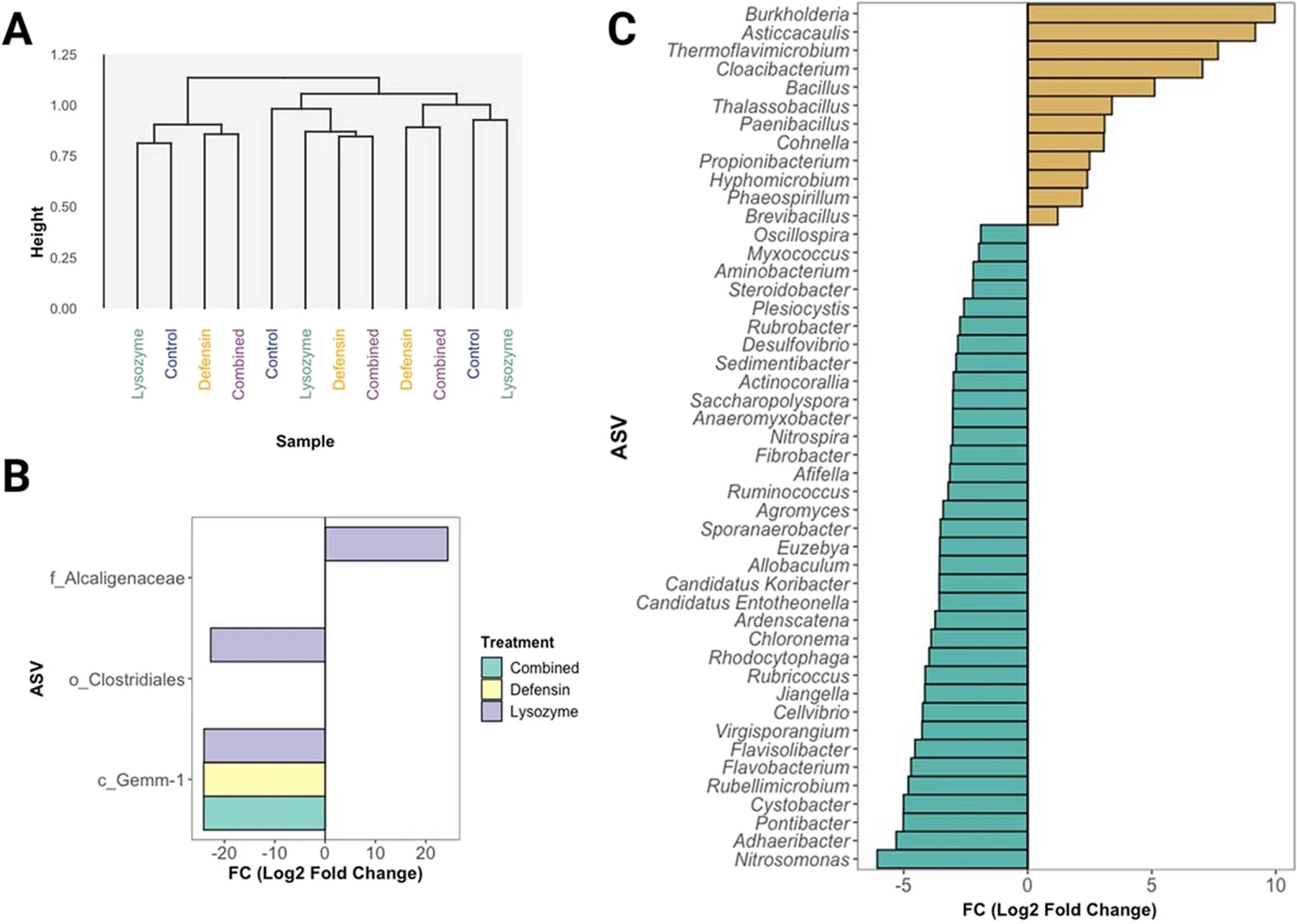
Differential abundance analysis of the rhizospheric soil bacterial community in *C. aurantifolia* expressing antimicrobials in phloem. Dendrogram based on ASVs of rhizospheric soil samples from plants overexpressing phloem-targeted antimicrobials (**A**). Differential abundance in the rhizospheric soil of *C. aurantifolia* plants overexpressing phloem-directed antimicrobials (lysozyme, defensin, or the combined antimicrobial treatment) compared with the control group (**B**). Differential abundance in roots versus rhizospheric soil samples of *C. aurantifolia* representing the FC values of enriched (brown) or suppressed (blue) genera in the roots compared to the soil (**C**)

Subsequently, we analyzed the microbiota relative abundance in rhizospheric soil samples from plants overexpressing phloem-directed antimicrobials. While no visual differences were detected at the phylum level (Fig. 1C), an apparent relative abundance increase in *Pseudomonas* and *Cellvibrio* was observed in the combined antimicrobial treatment compared to the control samples (Fig. 1D). However, statistical analysis showed no differences in relative abundance at the genus level. These findings aligned with the cluster analysis of the samples considering the presence and abundance of ASVs (Fig. 2A). Nevertheless, statistical changes were identified in other taxonomic categories: a relative abundance increase of an ASV from the Alcaligenaceae family in the lysozyme treatment (log2FC = 24.38, *p*-adj = 4.12 × 10^−13^), a relative abundance decrease in the ASV of the Clostridiales order with the same treatment (log2FC =  − 22.75, *p*-adj = 1.34 × 10 × 10^−4^), and a relative abundance reduction in the ASV of the Gemm-1 class in all three experimental treatments compared to the control (log2FC_lysozyme_ =  − 24.07, *p*-adj_lysozyme_ = 3.44 × 10^−5^; log2FC_defensin_ =  − 24.13, *p*-adj_defensin_ = 6.36 × 10^−5^; log2FC_combined_ =  − 24.13, *p*-adj_combined_ = 6.36 × 10^−5^) (Fig. 2B).

In addition, we identified 11 genera enriched in citrus roots compared to rhizospheric soil samples: *Burkholderia*, *Asticcacaulis*, *Thermoflavimicrobium*, *Cloacibacterium*, *Bacillus*, *Thalassobacillus*, *Paenibacillus*, *Cohnella*, *Propionibacterium*, *Hyphomicrobium*, and *Brevibacillus*. Conversely, 37 genera exhibited decreased abundance: *Oscillospira*, *Myxococcus*, *Aminobacterium*, *Steroidobacter*, *Plesiocystis*, *Rubrobacter*, *Desulfovibrio*, *Sedimentibacter*, *Actinocorallia*, *Saccharopolyspora*, *Anaeromyxobacter*, *Nitrospira*, *Fibrobacter*, *Afifella*, *Ruminococcus*, *Agromyces*, *Sporanaeronacter*, *Euzebya*, *Allobaculum*, Candidatus *Koribacter*, Candidatus *Entotheonella*, *Ardenscatena*, *Chloronema*, *Rhodocytophaga*, *Rubricoccus*, *Jiangella*, *Cellvibrio*, *Virgisporangium*, *Flavisolibacter*, *Flavobacterium*, *Rubellimicrobium*, *Cystobacter*, *Pontibacter*, *Adhaeribacter*, and *Nitrosomonas* (Fig. 2C).

### Alpha Diversity

**Fig. 3:**
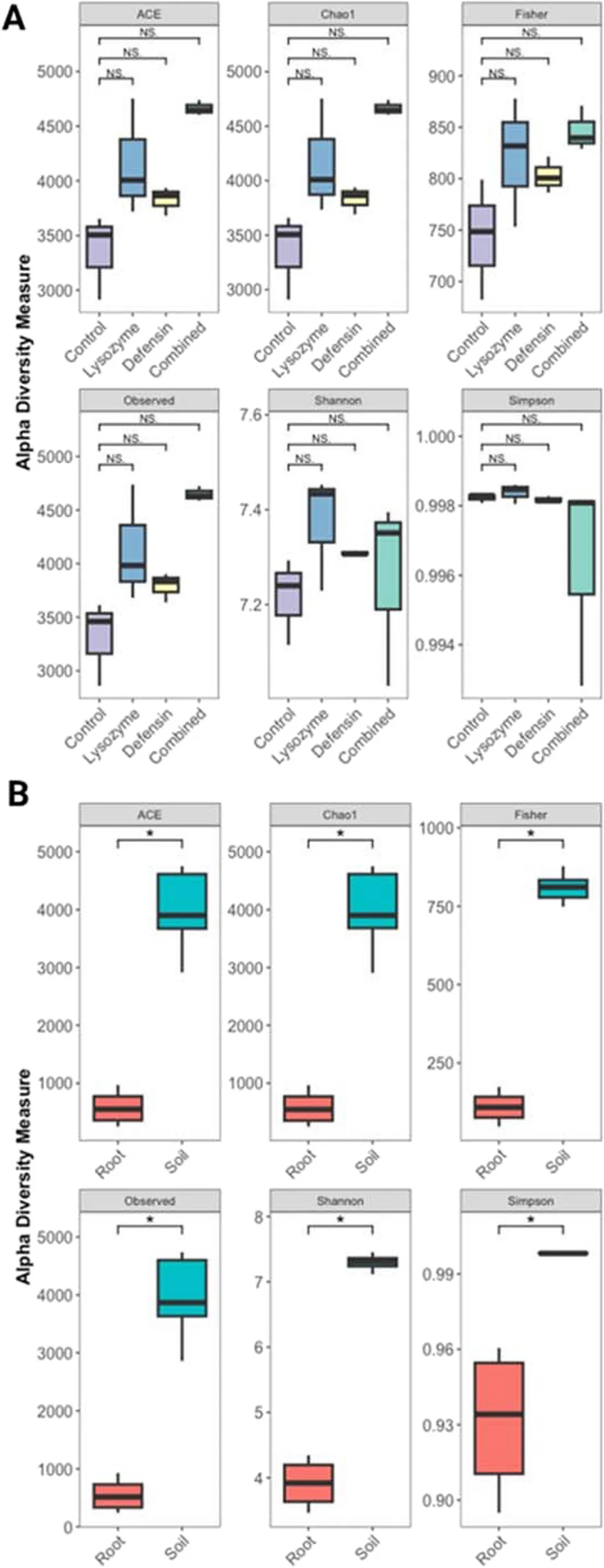
Differential abundance analysis of the bacterial community of *C. aurantifolia* overexpressing phloem-directed antimicrobials. Alpha diversity indices of rhizosphere soil bacterial communities of *C. aurantifolia* overexpressing phloem-directed antimicrobials (**A**). Comparison of alpha diversity indices in rhizospheric soil and root samples of *C. aurantifolia* (**B**). Statistical significance was assessed using the Wilcoxon rank-sum test (ns = *p*-value > 0.05; *p-value < 0.05)

Alpha diversity assessment using ACE, Chao1, Fisher, observed ASVs, Shannon, and Simpson indices were used to compare rhizospheric soil samples of the control group and plants overexpressing phloem-directed antimicrobials. Statistical analyses showed no significant differences between treatments (*p* > 0.05) (Fig. 3A). However, contrasting results were observed when comparing rhizospheric soil and roots, where statistically significant differences were found (*p* < 0.05, for all indices). Observed ASV, ACE, and Chao1 indices were higher in soil compared with roots (observed ASV, *μ*_soil_ = 3971 ± 589, *μ*_root_ = 550 ± 310; ACE, *μ*_soil_ = 4001 ± 575, *μ*_root_ = 580 ± 321; Chao1, *μ*_soil_ = 4004 ± 575, *μ*_root_ = 577 ± 321). The Fisher index showed higher abundance in the soil (*μ*_soil_ = 803 ± 55, *μ*_root_ = 110 ± 55). Additionally, the Shannon index displayed nearly double the value in soil (*μ*_soil_ = 7.3 ± 0.1, *μ*_root_ = 3.9 ± 0.4), and the Simpson index showed low dominance (*μ*_soil_ = 0.998 ± 0.157, *μ*_root_ = 0.931 ± 0.031), suggesting higher species diversity and homogeneity in rhizospheric soil samples (Fig. 3B).

### Beta Diversity Analysis

**Fig. 4:**
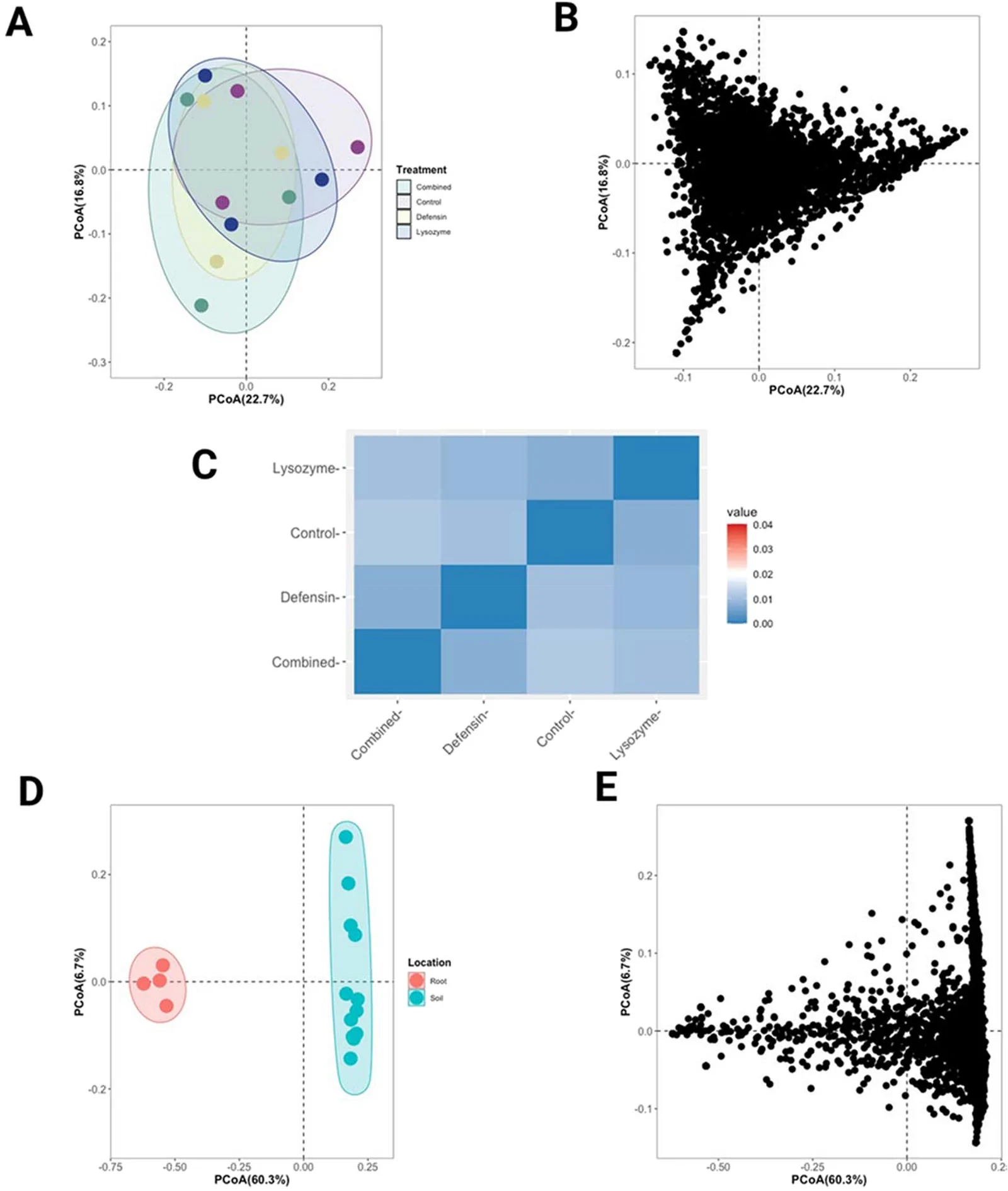
Beta diversity analysis of bacterial communities in *C. aurantifolia* expressing phloem-directed antimicrobials. PCoA of rhizospheric soil samples of plants overexpressing phloem-directed antimicrobials depicting sample distribution in two-dimensional space (**A**). PCoA showing the distribution of ASVs in two-dimensional space (**B**). Heatmap based on the UniFrac distance matrix from rhizospheric soil samples of plants overexpressing phloem-directed antimicrobials (**C**). PCoA of rhizospheric soil and root samples of *C. aurantifolia* depicting the sample distribution in two-dimensional space (**D**). PCoA of rhizospheric soil and root samples of *C. aurantifolia* showing the distribution of ASVs in a two-dimensional space (**E**)

We conducted a weighted UniFrac dissimilarity assessment to evaluate beta diversity considering species composition and relative abundance. PCoA was conducted using the distance matrix generated by weighted UniFrac. PCoA revealed overlaps among treatments and the ASVs were distributed in a shape akin to a wide triangle within a reduced-dimensionality space (Fig. 4A and B). These findings were supported by PERMANOVA indicating no statistical differences between treatments (*F* = 1.0091, *p* = 0.449) (Fig. 4C). Similarly, ANOSIM did not detect significant differences in composition or structure between groups (*R* =  − 0.05556, *p*-value = 0.647). However, comparing root and rhizospheric soil samples revealed group separation in PCoA, with taxa asymmetrically distributed according to density, particularly congregated towards the right, where rhizosphere soil samples were grouped (Fig. 4D, E). Statistical analysis via PERMANOVA confirmed significant differences between root and rhizospheric soil samples (*F* = 29.672, *p* = 0.001).

### Phylogenetic Diversity

We employed Faith Phylogenetic Diversity Index to quantify phylogenetic diversity, as it incorporates the sum of the branch lengths of the phylogenetic tree, providing a deeper understanding of biodiversity beyond UniFrac, PERMANOVA, or PCoA. This index effectively assesses the extent of evolutionary history represented within a community, assuming that greater phylogenetic diversity implies a wider span of accumulated evolutionary history among ASVs. This index is meaningful because higher phylogenetic diversity is related to increased ecosystem functionality, stability, and resilience [32].

We assessed the normal distribution of Faith index data and observed normality for treatments with lysozyme, β-defensin-2, and their combination, but not for the control (*W* = 0.75347–0.98716, *p* = 0.007674–0.7831). Therefore, we employed the Kruskal–Wallis test to assess statistical differences in phylogenetic diversity among treatments. Results indicated no significant differences in phylogenetic diversity among treatments as well as species richness (Table S2). Furthermore, comparing phylogenetic diversity between rhizospheric soil and root samples revealed significant differences, with soil samples exhibiting higher phylogenetic diversity than root samples (Table S3).

### Functional Prediction Analysis

**Fig. 5:**
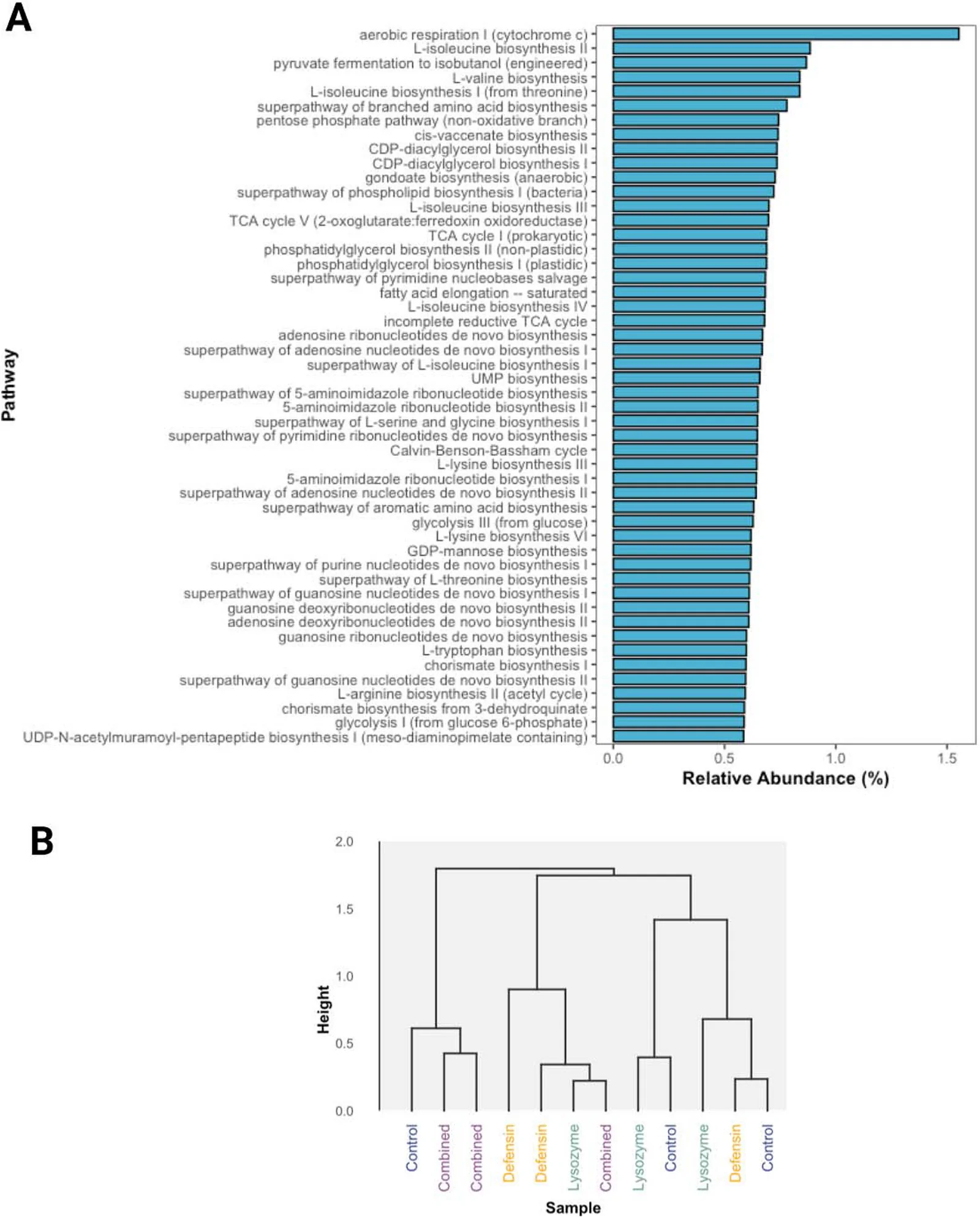
Functional prediction analysis of metabolic pathways. Bar chart of the global top 50 metabolic pathways identified in the rhizospheric soil of *C. aurantifolia* plants overexpressing phloem-directed antimicrobials (**A**). Clustering dendrogram of rhizospheric soil samples of *C. aurantifolia* plants overexpressing phloem-directed antimicrobials based on metabolic pathway abundance (**B**)

**Fig. 6:**
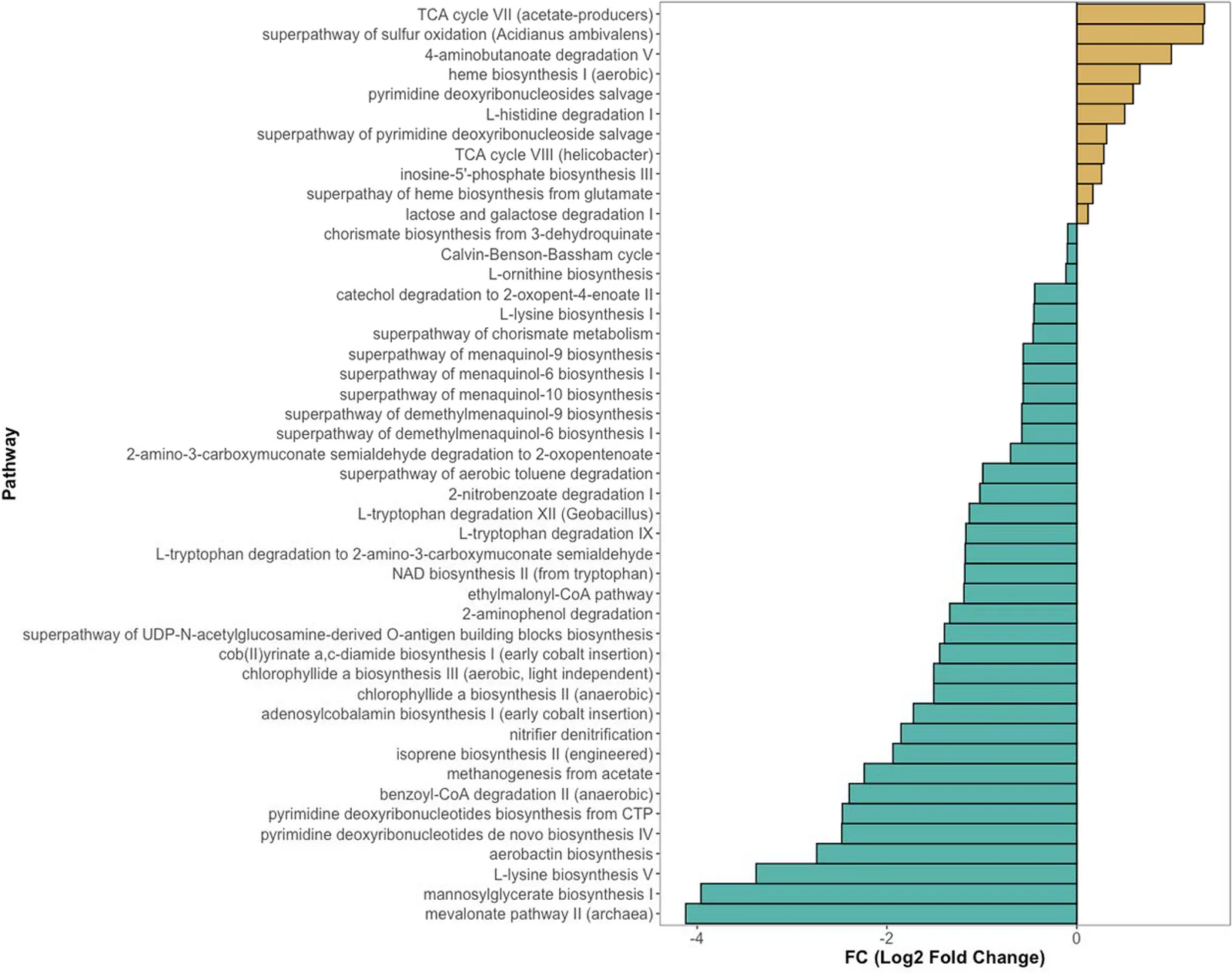
Comparative analysis of predicted metabolic pathway abundance in roots and rhizospheric soil of *Citrus aurantifolia* plants. The bars represent the FC values of enriched (brown) or suppressed (blue) metabolic pathways in the root compared to the soil of *C. aurantifolia* plants

Picrust2 analysis identified 431 metabolic pathways in rhizospheric soil samples, highlighting the prevalence of primary metabolic pathways such as aerobic respiration, pyruvate fermentation, biosynthesis of amino acids, nucleotides, and lipids as the most abundant (Fig. 5A). Statistical comparisons of these pathways showed no significant differences between treatments and control groups (Fig. 5B). However, comparing root and soil samples revealed 11 increased and 35 decreased metabolic pathways in roots compared to rhizospheric soil (Fig. 6). The pathways with the highest abundance in roots were TCA cycle VII (acetate-producers), superpathway of sulfur oxidation, 4-aminobutanoate degradation V, heme biosynthesis, pyrimidine deoxyribonucleoside salvage, L-histidine degradation I, superpathway of pyrimidine deoxyribonucleoside salvage, TCA cycle VIII, inosine-5′-phosphate biosynthesis III, and lactose and galactose degradation I. Downregulated pathways included superpathways including biosynthesis and degradation of amino acids, carbohydrates, carboxylic acids, aromatic compounds, generation of precursor metabolites and energy, inorganic nutrient metabolism, and the biosynthesis of cofactors, nucleotides, nucleosides, carriers, vitamins, metabolic regulators, and secondary metabolites.

## Discussion

The presence of specific bacterial taxa in soil samples insights into the physicochemical properties of the soil and ecological interactions within this environment [33]. Genera such as *Euzebya*, *Halomonas*, *Halorhodospira*, *Halothiobacillus*, *Marinibacillus*, *Marinimicrobium*, *Oceanobacillus*, *Rubellimicrobium*, *Salinicoccus*, *Salisaeta*, *Thalassobacillus*, and *Virgibacillus* suggest moderate salinity (Supplementary File 1), consistent with the soil of this geographic area [34–37]. Our experimental plantation is less than 10 km from the Pacific coast in Colima state and less than 2 km from the Armería River. This proximity explains the presence of bacteria that can be found in brackish conditions. Additionally, the presence of genera adapted to high temperatures such as *Thermoflavimicrobium*, *Geobacillus*, *Thermobacillus*, *Bacillus*, *Clostridium*, and *Thermoactinomyces* (Supplementary File 1) is in agreement with the high temperatures recorded in this geographical location (annual mean temperature 26.6 °C, with highs above 40 °C) [38, 39].

Differential abundance analysis revealed no statistically significant differences at the genus level in rhizosphere soil samples from *C. aurantifolia* plants overexpressing phloem-targeted antimicrobials. However, we identified three ASVs assigned to taxonomic categories above the genus level that exhibited differential abundance compared to the control group. The nature of these ASVs remained uncertain; it is unclear whether they are spurious assignments or represent differences in uncharacterized ASVs. In our study, out of 9668 identified ASVs without considering specific taxonomic assignments, these three non-genus-assigned ASVs constituted only 0.031% of the total number of differentially expressed ASVs. This low percentage supported the notion that treatments had minimal effects on the differential abundance of bacterial communities.

Proteobacteria, Actinobacteria, Acidobacteria, and Bacteroidetes are central components of the citrus microbiome [40]. Additionally, *Burkholderia*, *Asticcacaulis*, *Thermoflavimicrobium*, *Cloacibacterium*, *Bacillus*, *Thalassobacillus*, *Paenibacillus*, *Cohnella*, *Propionibacterium*, *Hyphomicrobium*, and *Brevibacillus* have been previously described as endophytes in citrus and other plant species [41–44], which is consistent with our results.

Regarding alpha diversity, overexpression of phloem-targeted antimicrobials exhibited no differences in observed richness and diversity indices (ACE, Chao1, Fisher, Observed ASVs, Shannon, and Simpson) compared to the control group. This absence of significant differences suggested an undetectable impact on the structure of the bacterial communities, specifically in terms of species richness, relative abundance, diversity, and evenness. Conversely, statistical analyses revealed significant differences between the root and rhizospheric soil niches, with the latter showing increased species richness, diversity, and evenness. In this context, several studies have shown that endophytic and rhizospheric bacterial communities are significantly influenced by the presence, abundance, and composition of root exudates, accounting for the observed differences between rhizosphere soil communities and root endophytes [45].

In beta diversity analysis, PCoA did not reveal differentiated groupings among plants overexpressing phloem-directed antimicrobials. This evidence was supported by PERMANOVA, which also indicated the absence of significant differences. However, we identified differences between the rhizospheric soil and root groups, consistent with previous reports highlighting these environments as distinct based on dimensional reduction analysis. Similar results were observed in the phylogenetic diversity analysis.

The utilization of PICRUSt2 for functional inference of the microbial community from 16S rRNA amplicon sequencing data enables the prediction of potential metabolic functionality based on taxonomic composition [46]. However, these predictions do not substitute data acquired through direct metagenomic analysis. Metagenomic analyses offer precise and non-predictive information regarding the functions of the microbial community, resulting in a more reliable and detailed characterization.

The assessment of the effects of antimicrobials on the bacterial community was conducted at one sampling time. Thus, long-term monitoring of microbial communities will be helpful to identify potential seasonal changes or assess whether the time elapsed between planting and evaluation is enough to detect antimicrobial effects on bacterial communities. In our study, amplicons were generated from the V3-V4 region of the 16S rRNA gene in rhizosphere soil and root samples, offering broad coverage of bacterial communities and comparability with existing research [47]. While effective, this approach has limitations, including reduced taxonomic resolution for certain species [48] and potential overestimation of abundance due to the multicopy nature of the 16S gene in some taxa [49]. However, the presence of multiple copies of this gene is uniform across samples, suggesting that the observed differences would be compensated. Other sequencing technologies, such as Oxford Nanopore and PacBio, can analyze the entire 16S gene and provide higher taxonomic resolution and a more comprehensive view of microbial diversity, highlighting the trade-offs between broad community coverage and detailed taxonomic insight [50].

Additionally, potential sources of error in our methodological approach included biases introduced during sample collection, presence of contaminant DNA, sample storage, DNA extraction, primer choice, PCR amplification, library preparation, sequencing platform, bioinformatic pipeline, and taxonomic classification [51] In addition, by using PCR-independent techniques, such as shotgun metagenomics, we could obtain more comprehensive insights into microbial communities by sequencing all genetic material, thus avoiding PCR and 16S rRNA-related biases. This approach allows for the identification of a broader range of microbial taxa, including viruses, fungi, and archaea, and enables functional analysis of microbial communities [52].

In general, our results showed that the expression or targeting strategy of the antimicrobial human lysozyme and human β-defensin-2 fused to a vascular-directed carrier protein (PP16) did not affect soil microbiome diversity. Lysozyme (E.C. 3.2.1.17) breaks the β-1–4 bonds between N-acetylglucosamine and N-acetylmuramic acid in bacterial cell walls [53]. Interestingly, some plant lysozymes can also hydrolyze chitin, albeit less efficiently [54]. Additionally, overexpression of egg lysozyme in cotton (*Gossypium hirsutum*) can control *Verticillium dahliae*, suggesting that an α-helix at the C-terminus of lysozymes may interfere with cell membranes and trigger innate defense responses [55]. Furthermore, some defensins exhibit antifungal activity by destabilizing cell membranes [56]. In fact, human β-defensin-2 controls *Candida albicans* by binding to phosphatidylinositol 4,5-bisphosphate in the cell membranes [57]. Therefore, it would be relevant to evaluate the impact of phloem-targeted antimicrobials on rhizosphere soil fungal communities.

The overexpression of these translational fusions was constitutive and strong, driven by the CaMV 35S promoter. However, symplasmic movement towards the vascular tissue occurs, allowing long-distance transport within the plant via phloem sieve elements. Transgenic Mexican lime (*C. aurantifolia*) plants were grafted onto bitter orange (*C. aurantium*) rootstocks, making the aerial part of the phloem a primary source of translationally fused antimicrobial agents. Nonetheless, the potential for symplasmic movement of antimicrobial proteins from the phloem to the cortex and root epidermis exists, resulting in the secretion of fused proteins from exudates that are abundant in citrus roots. However, these effects were not statistically significant in rhizosphere soil, according to the performed analysis. Overall, our results indicate that the approach employed confined the antimicrobial effect on vascular tissue, minimizing its impact on the rhizosphere soil microbiome.

## Conclusions

The analysis of the impact of GM citrus trees expressing phloem-directed antimicrobials on the soil microbiome demonstrated that the use of human lysozyme and β-defensin-2 fused to CsPP16 did not significantly alter the microbial diversity in the rhizosphere soil, suggesting a negligible ecological effect of this strategy on non-target soil microbiota. This finding supports the potential of phloem-targeted antimicrobials as a sustainable approach to managing citrus vascular diseases, such as Huanglongbing.

## Supplementary Information

Below is the link to the electronic supplementary material.

Supplementary file1 (XLSX 787 KB)

Supplementary file2 (DOCX 18 KB)

## Data Availability

Research data supporting this publication are available from the Short Read Archive (SRA) of the National Center for Biotechnology Information (NCBI) with Accession No PRJNA1084113.
