# Supplementary material for: Metagenomic Analysis of Rhizospheric Bacterial Community of Citrus Trees Expressing Phloem-Directed Antimicrobials
Source: Microb Ecol. 2024 Jul 15;87(1):93. doi: 10.1007/s00248-024-02408-w (PMC11249458; doi:10.1007/s00248-024-02408-w)
Supplement: Supplementary file 2 — Supplementary file2 (DOCX 18 KB) [file 248_2024_2408_MOESM2_ESM.docx]

**Supplementary Material**

**Table S1. 16S rRNA metagenomic sequencing statistics.**

| **Location** | **Treatment** | **Raw Reads** | **Filtered Reads** | **Denoised**  **Reads** | **Non-chimeric Reads** | **Assigned Bacterial Reads** |
| --- | --- | --- | --- | --- | --- | --- |
| Rizospheric Soil | Control | 99,560 | 86,207 | 84,416 | 75,750 | 74,325 |
|  | Control | 96,861 | 82,246 | 80,614 | 73,094 | 71,905 |
|  | Control | 60,103 | 51,707 | 50,277 | 44,626 | 44,131 |
|  | Lysozyme | 132,340 | 114,184 | 111,979 | 99,883 | 98,209 |
|  | Lysozyme | 129,281 | 111,107 | 108,958 | 99,303 | 98,039 |
|  | Lysozyme | 260,964 | 221,434 | 217,192 | 193,266 | 191,026 |
|  | β-Defensin-2 | 89,955 | 77,238 | 75,532 | 69,146 | 67,034 |
|  | β-Defensin-2 | 128,266 | 108,510 | 106,365 | 95,651 | 93,176 |
|  | β-Defensin-2 | 153,591 | 127,694 | 125,380 | 112,228 | 110,788 |
|  | Combined | 312,836 | 261,477 | 257,394 | 233,019 | 230,380 |
|  | Combined | 270,165 | 236,189 | 232,347 | 209,886 | 207,335 |
|  | Combined | 232,340 | 199,234 | 195,803 | 177,829 | 175,642 |
| Root | Control | 261,912 | 213,148 | 212,419 | 203,073 | 35,482 |
|  | Control | 390,635 | 350,184 | 349,696 | 341,981 | 6,295 |
|  | Control | 293,872 | 263,401 | 262,930 | 257,855 | 4,779 |
|  | Control | 350,674 | 314,366 | 313,700 | 304,718 | 18,770 |

**Table S2. Phylogenetic diversity of rhizospheric soil samples from citrus plants overexpressing phloem-targeted antimicrobials.**

| Treatment | Phylogenetic Diversity (PD) | | Species Richness (SR) | |
| --- | --- | --- | --- | --- |
|  | Mean ± SD | Chi-squared  (p-value) | Mean ± SD | Chi-squared  (p-value) |
| Control | 111.76±6.41 | 6.3846 (p=0.09433) | 1869.67±100.33 | 4.8462  (p=0.1834) |
| Lisozyme | 122.43±2.64 |  | 2054.67±118.57 |  |
| Defensin | 117.62±0.88 |  | 2007.33±35.50 |  |
| Combined | 117.83±5.18 |  | 2026.67±56.92 |  |

**Table S3. Phylogenetic diversity of rhizospheric soil and root samples from citrus plants.**

| Location | Phylogenetic Diversity (PD) | | Species Richness (SR) | |
| --- | --- | --- | --- | --- |
|  | Mean ± SD | Chi-squared  (p-value) | Mean ± SD | Chi-squared  (p-value) |
| Root | 44.91±9.47 | 8.4706  (p=0.003609) | 411.50±142.32 | 8.4706  (p=0.003609) |
| Soil | 117.93±4.31 |  | 1990.58±98.67 |  |
